# Mechanical Model of Tensile Loading of Geotechnical Reinforcement Materials

**DOI:** 10.3390/ma18020241

**Published:** 2025-01-08

**Authors:** Hao Liu, Zhen Zhang, Zuhui Long, Bin He, Feng Chen, Ziang Chen, Yuliang Lin

**Affiliations:** 1 Yueyang Planning, Survey and Design Institute Co., Ltd., Yueyang 414004, China; liuhao112024@163.com (H.L.); army06@163.com (Z.L.); hbhbhebin@sina.com (B.H.); chenfeng112024@163.com (F.C.); 2 School of Civil Engineering, Central South University, Changsha 410075, China; fighting036@163.com (Z.Z.); 15274956552@163.com (Z.C.)

**Keywords:** geotechnical reinforcement material, geogrid, gabion mesh, mechanical model, tensile loading

## Abstract

To reveal the mechanical behavior and deformation patterns of geotechnical reinforcement materials under tensile loading, a series of tensile tests were conducted on plastic geogrid rib, fiberglass geogrid rib, gabion steel wire, plastic geogrid mesh, fiberglass geogrid mesh, and gabion mesh. The full tensile force–strain relationships of the reinforcement materials were obtained. The failure modes of different geotechnical reinforcement materials were discussed. The standard linear three-element model, the nonlinear three-element model, and the improved Kawabata model were employed to simulate the tensile curves of the various geotechnical reinforcement materials. The main parameters of the tensile models of the geotechnical reinforcement materials were determined. The results showed that a brittle failure occurred in both the plastic geogrid rib and the fiberglass geogrid rib subjected to tensile loading. The gabion steel wire presented obvious elastic–plastic deformation behavior. The tensile resistance of fiberglass geogrid mesh was higher compared to that of plastic geogrid, which was mainly caused by the difference in the cross-sectional areas of these two types of geogrid. Due to a hexagonal mesh structure of gabion mesh, there was a distinct stress adjustment during the tensile process, resulting in a sawtooth fluctuation pattern in tensile curve. Compared to the strip geogrid material, hexagonal-type gabion mesh could withstand higher tensile strain and had greater tensile strength. Brittle failure occurred in both the plastic geogrid rib and the fiberglass geogrid rib when subjected to tensile loading. The gabion steel wire presented obvious elastic–plastic deformation behavior. The standard linear and nonlinear three-element models as well as improved Kawabata model could all well reflect the tensile behavior of geotechnical reinforcement materials before the failure of the material.

## 1. Introduction

Geotechnical reinforcement materials, such as geogrids and gabion mesh, are widely used to reinforce different kinds of geotechnical structures [1,2], which are well known as reinforced soil technologies [3,4,5]. When reinforcement materials are laid in the soil, the shear strength and tensile strength of the soil can be significantly improved. The reinforcement material interacts with the soil mass based on friction, interlocking, and embedding effects. Subsequently, the reinforcement material combines with the soil mass as a composite structure. The mechanical behavior of the reinforced soil body has the advantages of both the reinforcement material and the soil body, which stabilizes geotechnical structures [6,7,8,9]. Unlike other retaining structures [10,11,12], the reinforced soil structure is generally regarded as a flexible geotechncial structure.

Generally, the tensile strength of soil mass is extremely small. To improve the mechanical behavior of reinforced soil, a high tensile strength is generally required for geotechnical reinforcement materials [13]. Consequently, the tensile mechanical properties of reinforcement materials are the most fundamental technical indices used for the engineering design of reinforced soil structures. The stress–strain characteristics of the reinforcement material directly affect the engineering properties of the reinforced soil structure. With regard to the tensile mechanical properties of geotechncial reinforcement materials, many scholars have conducted a large amount of research work [14,15,16]. Shinoda et al. [17] obtained the tensile mechanical properties of three types of geogrids (PET, PP, and HDPE) from a series of short-term isolated tensile tests. Perkins et al. [18] proposed a constitutive model for reinforced soil materials, and the effectiveness of the model was verified through experiments. Boisse et al. [19,20] carried out biaxial tensile tests on reinforced soil materials, and the tensile stiffness of the reinforcement was particularly discussed. Palmeira et al. [21] investigated the mobilization of the bearing capacity of reinforcement materials under different tensile loads. Wilson et al. [22] performed a simulation analysis of the tensile properties of reinforcement materials as well as the friction and bearing interaction between the soil and reinforcement materials. Meanwhile, some scholars conducted a series of studies on the performance characteristics of glass fiber composite subjected to dynamic loading [23,24,25]. Peterson et al. [26] and Shokrieh et al. [27] investigated the dynamic tensile properties of reinforced materials in different strain rate ranges.

An essential issue in engineering practice is considering how various factors influence the tensile characteristics of reinforcement materials [28,29,30]. Wesseloo et al. [31] analyzed the influence of the tensile rate on the tensile properties of HDPE geomembranes. Lifshitz et al. [32] analyzed the effect of the strain rate on the tensile mechanical behavior of angle ply glass/epoxy composites, and the results showed that strain rate did not affect the material’s elastic modulus. A similar conclusion was also drawn in the studies of Melin [33] and Ochola [34]. Subaida et al. [35] studied the effect of different material sizes on the tensile mechanical properties of reinforcement materials. Mendes et al. [36] analyzed the effects of a confined environment and the soil particle characteristics on the tensile mechanical properties of reinforcement materials.

Additionally, the tensile mechanical behavior under the coupled effects of strain rate and temperature has been examined by many researchers [37,38,39,40,41]. Shirinbayan et al. [42] analyzed the effects of two temperatures on the damage behavior of glass fiber composites from quasistatic tension to high-strain-rate tension. Hawileh et al. [43] studied the tensile strength and elastic modulus of glass fiber and hybrid composites at temperatures ranging from 25 to 300 °C, and the results indicated that the tensile strength and elastic modulus of the resin material exhibited high temperature sensitivity near the glass transition temperature. Zhang et al. [44] employed molecular dynamics’ simulation to analyze the interfacial behavior of glass fiber/PP composites under different loading temperatures and strain rates. Zhang et al. [45] investigated the effects of layup (the stacking order of laminates), strain rate, and high temperature on the mechanical behavior of carbon-fiber-reinforced thermoplastic composites in quasistatic and dynamic tests. Elanchezhian et al. [46] indicated that CFRP composites exhibited higher tensile and bending properties at different strain rates (1.5–2.5 mm/min) and temperatures (35–70 °C) compared to GFRP composites. Sato et al. [47] used finite element analysis to simulate the transverse tensile failure modes of UD carbon-fiber-reinforced plastics under different strain rates and temperatures, and the results indicated that the temperature-related softening effect reduced the stiffness of the composites during the loading process [48,49,50].

The existing research has effectively enhanced the understanding of the tensile properties of different geotechnical reinforcement materials for engineering technicians. However, it is noted that the mechanical properties of different geotechnical reinforcement materials vary significantly, and tensile tests on a specific given material are still required to determine its tensile characteristics. Based on tensile tests, selecting a suitable mechanical model is an engineering-relevant research topic to simulate the stress–strain curves of geotechnical reinforcement material and to further analyze its internal deformation characteristics.

Consequently, based on the reinforced soil slope project of the 4970 Special Line in Dangyang City, Hubei Province, China, this study carried out tensile tests on fiberglass geogrid, plastic geogrid, and gabion mesh to analyze the tensile mechanical properties of these reinforcement materials. Furthermore, attempts were made to fit the tensile curves of the reinforcement materials by using the standard linear three-element model, the nonlinear three-element model, and the improved Kawabata model to investigate the deformation characteristics of the reinforcement materials. This study aimed to provide some guidance for the application of geotechnical reinforcement materials in engineering practice and to offer a theoretical model for analyzing the mechanical properties of reinforcement materials subjected to tensile loading.

## 2. Materials and Methods

Geotechnical reinforcement materials are widely adopted to enhance the shear strength of soil. Generally, the tensile strength of soil is extremely low. As such, reinforcement materials are placed in the soil body to improve the tensile strength and shear strength of the reinforced soil. Consequently, tensile strength is a key parameter for geotechnical reinforcement materials.

### 2.1. Materials

**Figure 1:**
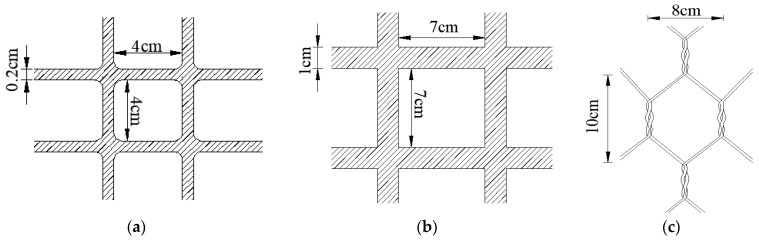
Dimensions of reinforcement material in tensile test. (**a**) Plastic fiber geogrid; (**b**) fiberglass geogrid; (**c**) gabion mesh.

Three different types of geotechnical reinforcement materials (i.e., plastic geogrid, fiberglass geogrid, and gabion mesh) were selected for tensile tests. The plastic geogrid was fabricated with horizontal and vertical ribs in a series of grids. The grid size of the plastic geogrid was 4 × 4 cm, with a rib width of 0.2 cm. The thickness of the plastic geogrid was 0.2 cm. The grid size of fiberglass geogrid was 7 × 7 cm, with a rib width of 1.0 cm. The thickness of the plastic geogrid was 0.1 cm. The gabion mesh was a kind of hexagonal mesh structure produced by twisting many gabion wires. As for each hexagonal gabion mesh, the longitudinal length was about 10 cm, and the transverse length was about 8 cm. The diameter of the gabion wire was 2.7 mm. The dimensions of the reinforcement material in the tensile test are shown in Figure 1.

### 2.2. Methods

**Figure 2:**
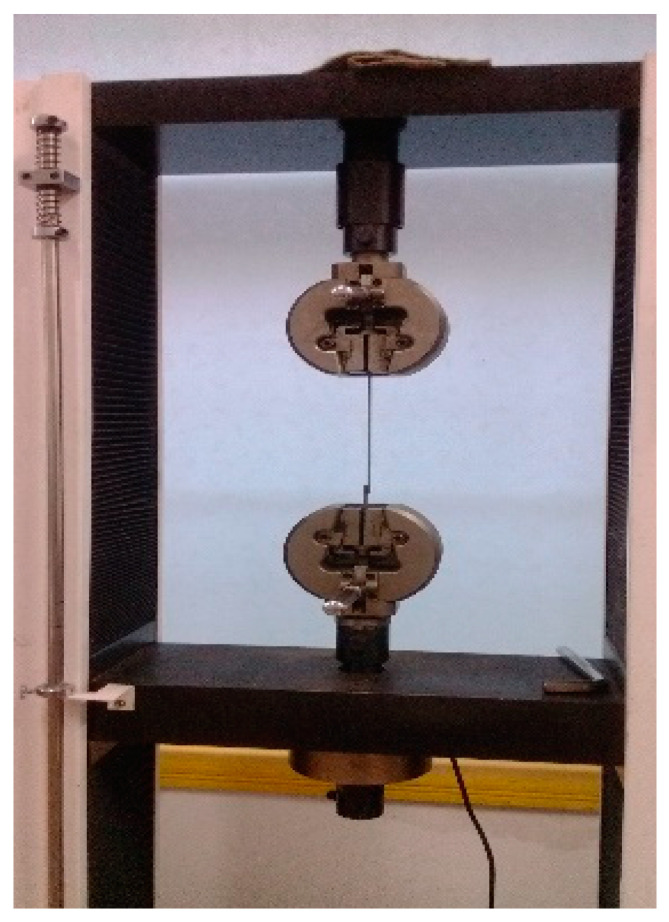
Tensile test of gabion steel wire.

Since the plastic geogrid consisted of a series of horizontal and vertical ribs, the tensile test was firstly conducted on a plastic geogrid rib and a fiberglass geogrid rib to obtain their basic tensile mechanical characteristics. A tensile test was also conducted on the gabion steel wire using special tensile equipment, as shown in Figure 2. Thereafter, the tensile tests were carried out on the three different types of geotechnical reinforcement mesh.

**Figure 3:**
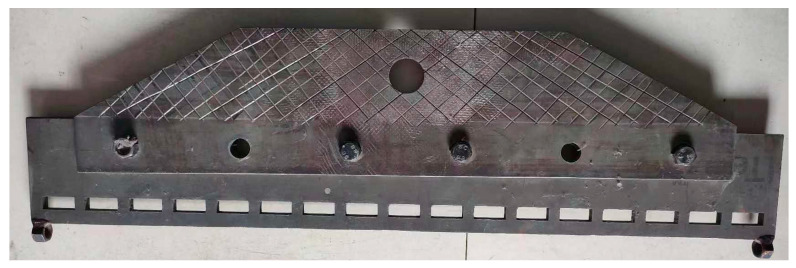
The special clamp used for the hexagonal gabion mesh.

Because the gabion mesh was a reinforcement material with hexagonal mesh within a certain width, a special clamp was required to hold the material in the tensile test, as shown in Figure 3. The clamp had many transverse clamping holes with different sizes, and, at the both ends of the clamp, there were lateral clamping holes, which could effectively limit the lateral deformation of the geotechnical reinforcement material. A pair of clamps were used to fix the top side and the bottom side of each gabion mesh to the tensile testing equipment.

**Figure 4:**
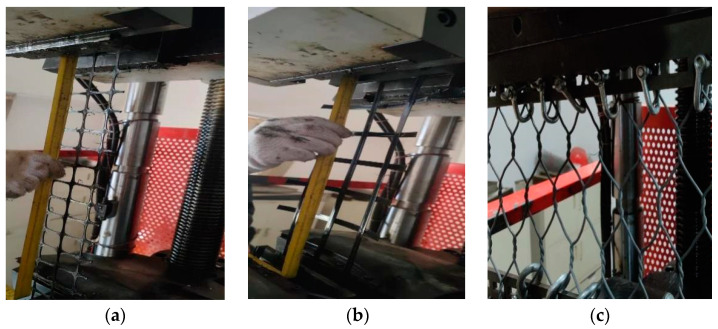
Tensile test on different types of geotechnical reinforcement materials. (**a**) Plastic geogrid mesh, (**b**) fiberglass geogrid mesh, and (**c**) gabion mesh.

Tensile tests were conducted on plastic geogrid mesh, fiberglass geogrid mesh, and gabion mesh by using a universal testing machine, as shown in Figure 4. The testing machine could provide both compressive and tensile forces within a range of 0–10 t. The tensile force and displacement could be directly collected automatically. Three parallel tests were performed for each material. As for the tensile test of the gabion mesh, due to the width limitation of the clamp in the universal testing machine, bolts were used to connect the clamp to the gabion, and then the clamp gripped the fixture for the tensile test. Unlike the gabion mesh, the geogrid mesh was prone to separating between the transverse and longitudinal ribs of the reinforcement material. Therefore, the geogrid mesh was clamped directly using plates for the tensile test, and the width of the geogrid in the test was determined based on the width of the clamp.

The tensile tests were all conducted at a room temperature of approximately 25 °C, with a tensile rate of about 2 mm/min. A series of parallel tensile tests were conducted for each geotechnical reinforcement material based on the requirements in the code (Technical Specifications for Application of Geosynthetics in Highway (JTG/T D32-2012)). The results of parallel tests indicated a small and acceptable experimental error.

## 3. Results

The mean values of the main tensile parameters of the different types of geotechnical reinforcement materials were obtained based on tensile tests, which are shown in Table 1. The tensile force at 2% or 5% strain refers to the tensile force when the tensile strain reached 2% or 5%. The maximum tensile strength was the tensile stress when the reinforcement material was damaged.

### 3.1. Reinforcement Rib Results

**Figure 5:**
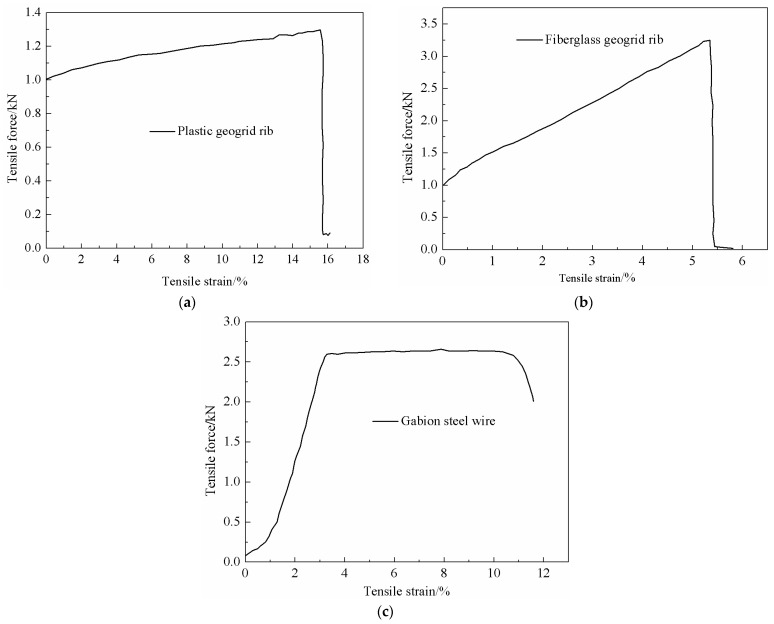
Typical tensile curves of (**a**) plastic geogrid rib, (**b**) fiberglass geogrid rib, and (**c**) gabion steel wire.

Figure 5 shows the typical tensile force–strain curves of the plastic geogrid rib, fiberglass geogrid rib, and gabion steel wire. The force–strain curves of the plastic geogrid rib and fiberglass geogrid rib exhibit similar trends. Initially, when the tensile strain was small, the tensile force increased linearly as the tensile strain increased, indicating a linear elastic characteristic. When the tensile strain reached a certain value, the geogrid rib fractured suddenly, and the tensile force rapidly decreased to zero. It was inferred that brittle failure occurred in both the plastic geogrid rib and the fiberglass geogrid rib when subjected to tensile loading. The values of the maximum tensile loading corresponding to the brittle failure of the plastic geogrid rib and fiberglass geogrid rib were 1.24 kN and 3.24 kN, respectively. The fiberglass geogrid rib was much stronger than the plastic geogrid rib. However, when referring to tensile strength, the difference between them was not so obvious. The tensile strength could also be obtained by dividing the maximum tensile loading by its area. It was determined that the values of the tensile strength of the plastic geogrid rib and fiberglass geogrid rib were 310 kPa and 314 kPa, respectively. The tensile strengths of the plastic geogrid rib and fiberglass geogrid rib were quite close.

As for the gabion steel wire, the characteristics of the stress–strain curve were different from those of the plastic geogrid rib or fiberglass geogrid rib. Initially, when the tensile strain was small, the tensile force increased gradually with the growth in strain, which indicated an elastic stage at a low strain level. Thereafter, when the strain reached a certain value (about 3.2%), a prolonged yield phase was observed in the gabion steel wire, which indicated a plastic characteristic. A long plastic stage was observed in the gabion steel wire, so a failure warning can be easily established when gabion steel wire is adopted in engineering practice.

The differences in the tensile characteristics among the plastic geogrid rib, the fiberglass geogrid rib, and the gabion steel wire were discussed. The maximum strain of the plastic geogrid rib is larger than that of the fiberglass geogrid rib, while the maximum tensile force of fiberglass geogrid rib is larger than that of plastic geogrid rib. If the deformation of reinforced soil is strictly required, fiberglass geogrid is recommended to reinforce the soil. Additionally, the tensile strength of gabion steel wire is the largest, and it presents obvious elastic–plastic deformation behavior. In engineering applications, it is essential to select the appropriate flexible reinforcement material based on the specific project requirements.

### 3.2. Reinforcement Mesh Results

**Figure 6:**
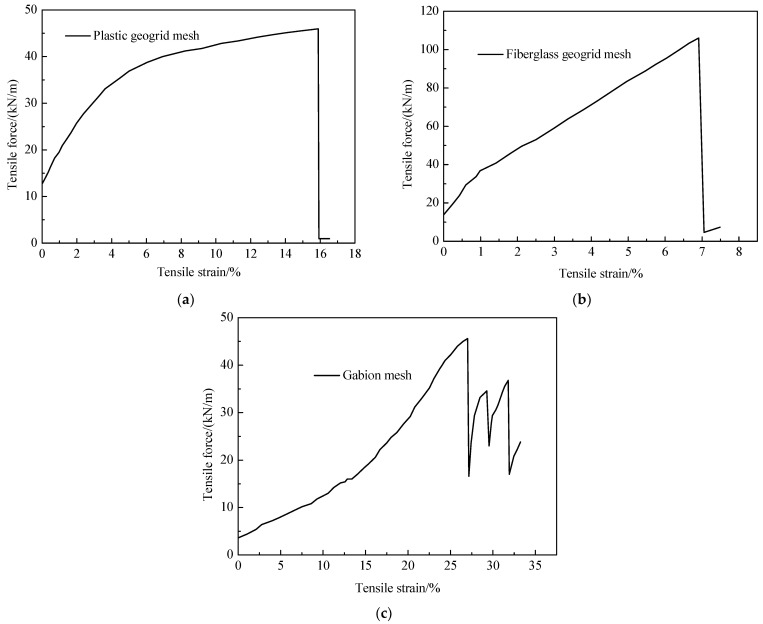
Typical tensile curves of (**a**) plastic geogrid mesh, (**b**) fiberglass geogrid mesh, and (**c**) gabion mesh.

Figure 6a,b show the typical tensile curves of the plastic geogrid mesh, fiberglass geogrid mesh, and gabion mesh. Considering the impacts of production methods on the properties of reinforcement materials, the test results obtained are only applicable to the materials selected for this test. Generally, the stress–strain curves of plastic geogrid mesh and fiberglass geogrid mesh were quite similar, although the nonlinear characteristics of the plastic geogrid mesh were more obvious than those of the fiberglass geogrid mesh. Apart from that, an additional difference was the superior toughness of the plastic geogrid mesh, which achieved a maximum elongation rate of 16.63%. The maximum elongation rate of the plastic geogrid mesh was significantly higher than that of the fiberglass geogrid mesh (i.e., 5.3%). The tensile resistance of the fiberglass geogrid mesh was higher compared to that of the plastic geogrid. The maximum tensile force of the plastic geogrid mesh was 1.24 kN, while it reached 3.14 kN for the fiberglass geogrid mesh. The differences in the tensile force were mainly caused by the differences in the cross-sectional areas of these two types of geogrid since the values of the tensile strength of the plastic geogrid rib and the fiberglass geogrid rib were quite close (see Table 1).

Figure 6c shows a typical tensile curve of the gabion mesh. It can be observed that due to the hexagonal mesh structure, there is a distinct stress adjustment process occurring throughout the entire gabion mesh during the tensile process, resulting in a sawtooth fluctuation pattern in the curve of tensile force versus strain. Specifically, when one of the wires reaches its ultimate strength and fractures, the gabion mesh experiences a significant drop in strength. However, the remaining wires quickly redistribute the stress state to bear the tensile load, which makes the tensile force gradually increase and finally form a series of periodic sawtooth fluctuations. Compared to the strip geogrid material, hexagonal-type gabion mesh can effectively withstand higher tensile strain and had increased tensile strength.

Compared with that of the fiberglass geogrid rib, a notable increase in the maximum tensile strain was observed for the fiberglass geogrid mesh. A similar phenomenon was also observed for the gabion mesh. For example, the maximum tensile strain of the fiberglass geogrid rib was 5.30%, and it was 7.23% for the fiberglass geogrid mesh, indicating that the grid structure significantly enhanced the flexibility of the geogrid materials. The maximum tensile strain of the gabion steel wire was just 11.52%, while the maximum tensile strain of the gabion mesh was higher, at 26.58%. The twisted hexagonal shape of gabion mesh makes it deformable when subjected to tensile loading, which increases the tensile strain that it can withstand significantly. The tensile strength of the fiberglass geogrid mesh was larger compared with that of the plastic geogrid mesh and gabion mesh, and it could reach 98.9 kN/m. The maximum tensile strength of the plastic geogrid mesh was about 39.8 kN/m, which was close to the maximum tensile strength of the gabion mesh of 43.5 kN/m.

### 3.3. Failure Model of Reinforcement Materials

**Figure 7:**
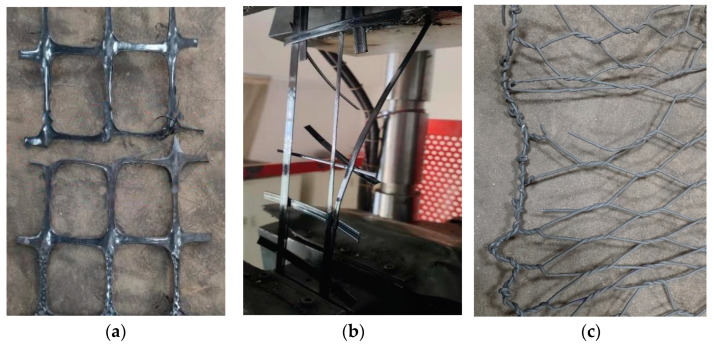
Failure models of different geotechnical reinforcement materials. (**a**) Plastic geogrid mesh, (**b**) fiberglass geogrid mesh, and (**c**) gabion mesh.

The failure modes of these three types of geotechnical reinforcement materials are shown in Figure 7. Failure was observed at the nodes of the plastic geogrid mesh during the tensile process. Maybe there was stress concentration in the node section, and the node was a weak part of the plastic geogrid mesh. The tensile strength of plastic geogrid mesh is basically determined by the strength of its nodes. The tensile strength at the nodes should be appropriately reinforced during the manufacturing process of plastic geogrids. Due to the sudden fracture at the nodes, brittle failure was observed in the plastic geogrid mesh.

The fiberglass geogrid rib fractured in the middle section when subjected to tensile loading, and the transverse ribs peeled away from the longitudinal ribs. It seemed that there was no stress concentration across the connection section of the ribs. Consequently, the tensile strength of each geogrid rib can be well utilized to determine the total tensile strength of fiberglass geogrid mesh. The tensile strength of the fiberglass geogrid was much higher than that of the plastic geogrid. Additionally, brittle failure was observed in the fiberglass geogrid mesh when subjected to tensile loading.

The edge wires compressed toward the center of the gabion mesh during the tensile process, and it gradually reached a yield stage when some of the wires broke. A bottleneck phenomenon was observed during the tensile test. According to the tensile test results for the gabion steel wire, large plastic deformation was observed. Consequently, with the increase in tensile strain, the gabion steel wires showed increased tensile load. There was no stress concentration due to the elastic–plastic characteristics of gabion steel wire. Meanwhile, the hexagonal shape of the gabion mesh made it more deformable. The maximum tensile strain of the gabion mesh (26.58%) was significantly larger than that of the plastic geogrid mesh (12.70%) or fiberglass geogrid mesh (7.23%).

## 4. Tensile Mechanical Model

It is of significant importance to develop a reasonable tensile mechanical model for geotechnical reinforcement materials for their engineering design and application. Based on the full force–strain curves obtained from the tensile test, this study adopted a standard linear three-element model, a nonlinear three-element model, and an improved Kawabata model to reveal the tensile characteristics of the geotechnical reinforcement materials. The main parameters I then tensile mechanical model can well reflect the physical characteristics of reinforcement materials.

### 4.1. Model Overview 

#### 4.1.1. Linear and Nonlinear Three-Element Model

**Figure 8:**
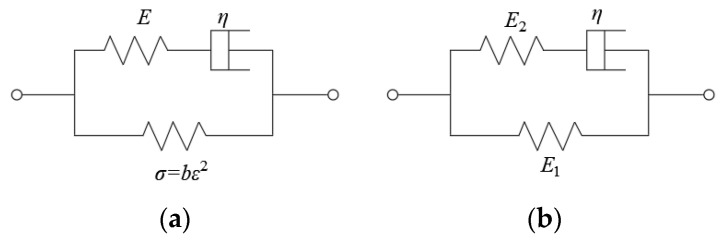
Tensile mechanical model. (**a**) Nonlinear three-element model and (**b**) standard linear three-element model.

The standard linear three-element model is composed of a Maxwell body and a linear spring body in parallel, and the nonlinear three-element model is composed of a Maxwell body and a spring body in parallel, as shown in Figure 8. The formulas for both the nonlinear and linear spring body can be expressed as

(1) $$\left\{\begin{array}{ll} \sigma =b\varepsilon^{2} & Nonlinear\\ \sigma =E_{1}\varepsilon & Linear \end{array}\right.$$

where *σ* represents the stress, *ε* refers to strain, and *b* is a parameter.

Based on the tensile mechanical model, the formula for the standard linear three-element model can be established as

(2) $$\frac{d\sigma }{dt}+\frac{E_{2}}{\eta }\sigma =(E_{1}+E_{2})\frac{d\varepsilon }{dt}+\frac{E_{1}E_{2}}{\eta }\varepsilon$$

The nonlinear model is

(3) $$\frac{d\sigma }{dt}+\frac{E_{0}}{\eta }\sigma =(E_{0}+2b\varepsilon )\frac{d\varepsilon }{dt}+\frac{E_{0}b}{\eta }\varepsilon^{2}$$

in which *E*_0_, *E*_1_, and *E*_2_ are the elastic modulus at different positions; *η* represents the coefficient of viscosity; *σ* and *ε* refer to stress and strain, respectively; and *b* is a parameter of nonlinear spring bodies, *σ* = *b*·*ε*^2^.

For the tensile test, we adopted a conventional constant-rate elongation test. Consequently, the tensile strain is proportional to the time *t*; that is, *ε* = *kt*. *k* is the coefficient of proportionality between strain *ε* and time *t*. Subsequently, *ε* = *kt* can be substituted into Equations (2) and (3):

(4) $$\frac{d\sigma }{dt}+\frac{E_{2}}{\eta }\sigma =(E_{1}+E_{2})k+\frac{E_{1}E_{2}}{\eta }kt$$

(5) $$\frac{d\sigma }{dt}+\frac{E_{0}}{\eta }\sigma =(E_{0}+2bkt)k+\frac{E_{0}b}{\eta }k^{2}t^{2}$$

The differential Equations (4) and (5) can be solved using a combination of the initial conditions (*t* = 0, *σ*_0_ = 0). Thereafter, the stress–strain formulas of these two three-element models can be obtained.

The standard linear model is

(6) $$\sigma =k\eta [1-\exp(\frac{E_{2}\varepsilon }{k\eta })]+E_{1}\varepsilon$$

The nonlinear model is

(7) $$\sigma =k\eta [1-\exp(-\frac{E_{0}\varepsilon }{k\eta })]+b\varepsilon^{2}$$

#### 4.1.2. Improved Kawabata Model

**Figure 9:**
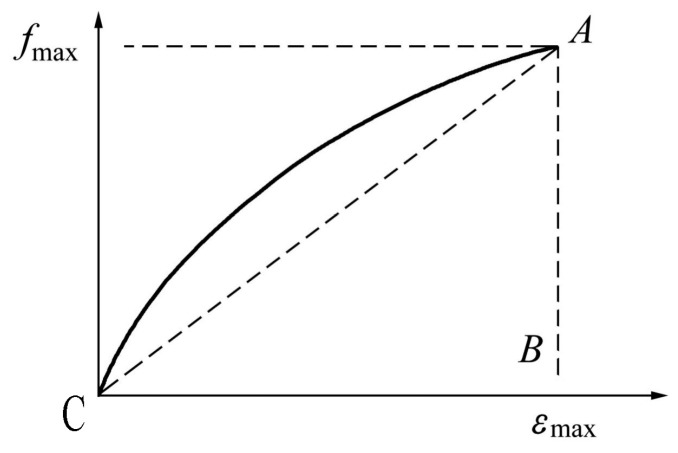
Improved Kawabata model used for tensile test.

The improved Kawabata model was adopted to reflect the tensile characteristics of the geotechnical reinforcement materials, as shown in Figure 9. The formula of the improved Kawabata model can be simplified as [18]

(8) $$f(\varepsilon )=\frac{{f_{\max}}^{2}\times {L_{t}}^{4}\times \varepsilon }{2W_{t}{\left[(L_{t}-1)\frac{L_{t}\times f_{\max}}{2W_{t}}\varepsilon +1\right]}^{3}}$$

where *f*_max_ is the tensile force corresponding to the maximum strain (fracture elongation); *W*_t_ is the tensile energy per unit area, and its value is equal to the area enclosed by the tensile curve of the reinforcement and horizontal axis; *L*_t_ is the tensile linear ratio, and its value can be determined as the ratio of *W*_t_ to the area of the triangle *OAB*. When the tensile curve is convex upward, *L*_t_ > 1; otherwise, *L*_t_ < 1.

### 4.2. Model Simulation and Analysis

The tensile characteristics of the different types of geotechnical reinforcement materials were analyzed, including those of the plastic geogrid rib, the fiberglass geogrid rib, the gabion steel wire, the plastic geogrid mesh, the fiberglass geogrid mesh, and the gabion mesh. The tensile force–strain curve before the failure of the reinforcement material is mainly discussed here.

**Figure 10:**
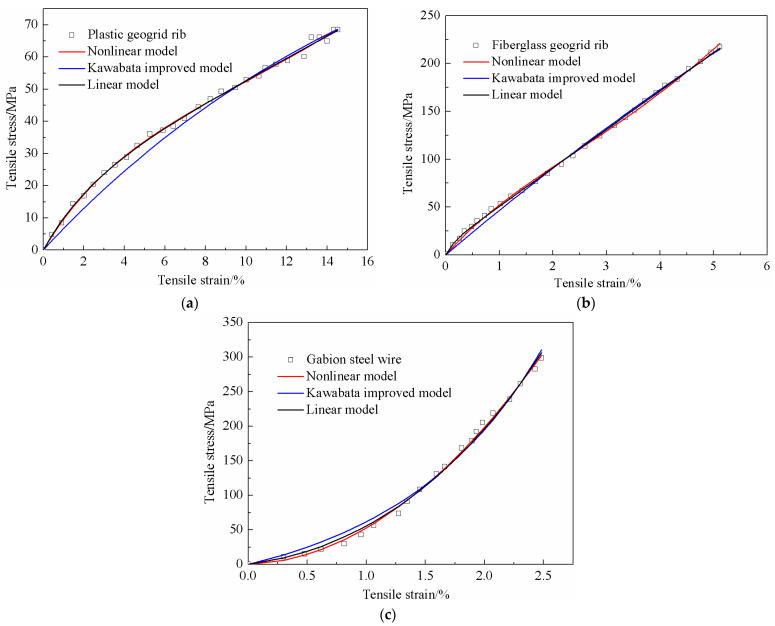
Simulation results of the tensile curves of different reinforcement ribs. (**a**) Plastic geogrid rib; (**b**) fiberglass geogrid rib; and (**c**) gabion steel wire.

The linear three-element model, the nonlinear three-element model, and the improved Kawabata model were used to simulate the tensile curves of the geotechnical reinforcement materials. The fitting parameters were obtained by using the least squares method. Table 2 shows the fitting parameters of the plastic geogrid rib, the fiberglass geogrid rib, and the gabion steel wire. The simulation results of the tensile curves for the three geotechnical reinforcement ribs are shown in Figure 10.

Both the linear *three-element* model and nonlinear *three-element* model well captured the tensile characteristics of the plastic geogrid rib. However, the improved Kawabata model did not accurately simulate the tensile curve in the low strain section. The tensile stress determined by the improved Kawabata model was a little lower than the test result. As for the fiberglass geogrid rib, the results obtained with these three models were quite consistent with the test data when the tensile strain was small (within a range of 0–9.2%). The tensile stress determined with the improved Kawabata model was also a little lower than the test result when the tensile strain was within the range of 0–1.5%. All in all, the linear *three-element* model and nonlinear three-element model could well simulate the tensile characteristics of both plastic geogrid ribs and fiberglass geogrid ribs. However, the simulation results obtained with the improved Kawabata model were worse than those obtained from the linear or nonlinear three-element model.

The nonlinear three-element model produced the best simulation effect regarding the tensile curve of gabion steel wire, followed by the linear three-element model, and the worst was the improved Kawabata model. The improved Kawabata model could not well capture the tensile characteristics of the gabion steel wire at the small tensile strain stage. The improved Kawabata model slightly overestimated the tensile stress in the low tensile strain range (0–1.3%).

**Figure 11:**
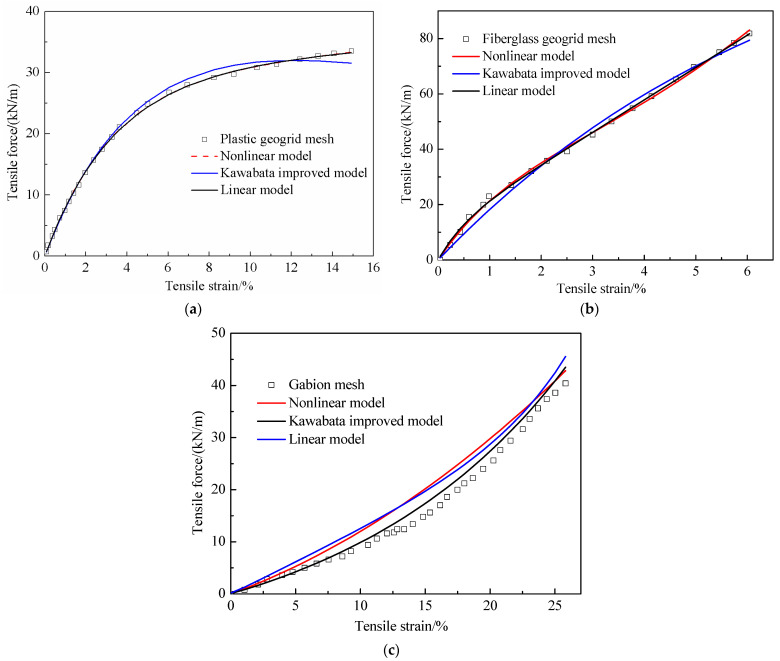
Simulation results of the tensile curves of different geotechnical reinforcement meshes. (**a**) Plastic geogrid mesh; (**b**) fiberglass geogrid mesh; (**c**) gabion mesh.

The fitting results of the plastic geogrid mesh, the fiberglass geogrid mesh, and the gabion mesh using the standard linear *three-element* model, nonlinear three-element model and Improved Kawabata model are shown in Table 3. The simulation results of the tensile curves of the three geotechnical reinforcement meshes are shown in Figure 11.

The simulation results obtained with the standard linear *three-element* model and nonlinear *three-element* model were very close for both the plastic geogrid mesh and fiberglass geogrid mesh. Both of them well simulated the tensile characteristics of both the plastic geogrid mesh and fiberglass geogrid mesh. However, the improved Kawabata model produced a poorer simulation result compared with the linear and nonlinear *three-element* models. The improved Kawabata model captured the tensile behavior of the plastic geogrid mesh at low tensile strains, but the fitting results worsened as the tensile strain increased. In the low tensile strain range, the improved Kawabata model underestimated the tensile force of the fiberglass geogrid mesh, while the model overestimated the tensile force in the high tensile strain range.

The standard linear *three-element* model, the nonlinear *three-element* model, and the improved Kawabata model all well captured the tensile characteristics of the gabion mesh before the gabion steel wire fractured. The simulation results of the improved Kawabata model were the best among these three analysis models. Apart from that, the force–strain curve of the gabion mesh exhibited a sawtooth shape when one of the gabion steel wires fractured. The above three analysis models could not reflect the tensile behavior of the gabion mesh after it fractured.

## 5. Discussion

Theoretically, the mechanical properties of flexible reinforcement materials exhibit nonlinear viscoelastic characteristics and a complex relaxation time spectrum, which reflect the molecular chain motion state and the internal deformation characteristics of a material [18,51]. However, to simplify the description of the viscoelastic behavior of such materials in practical engineering, the three-element model with an average relaxation time is commonly employed. The three-element model and the Kawabata modified model were adopted to describe the mechanical behavior of tensile reinforcements in this study, with results that are consistent with the findings of Nachean [52], Vangheluwe [53], and Wesseloo [31]. As for the Maxwell unit in the standard or nonlinear three-element models, an increase in parameter *η* indicates enhanced material elasticity. When *η* approaches infinity, the model exhibits the characteristics of a Hookean elastic solid, whereas a smaller value of *η* implies a greater flow deformation capability. Future research can be extended to the macromechanical properties of these reinforcements to enhance the understanding of reinforcement behavior and their mechanical models.

## 6. Conclusions

Brittle failure occurs in both the plastic geogrid rib and the fiberglass geogrid rib when subjected to tensile loading. The gabion steel wire presents obvious elastic–plastic deformation behavior. The maximum strain of the plastic geogrid rib is larger than that of the fiberglass geogrid rib, while the maximum tensile force of the fiberglass geogrid rib is larger than that of the plastic geogrid rib. The tensile strength of the gabion steel wire is the largest. In engineering application, it is essential to select the appropriate flexible reinforcement material based on the specific project requirements.

The tensile resistance of the fiberglass geogrid mesh is higher compared to that of plastic geogrids. The differences in tensile force are mainly caused by the differences in the cross-sectional areas of these two types of geogrid. Due to the hexagonal mesh structure, there is a distinct stress adjustment process throughout the entire gabion mesh during the tensile process, resulting in a sawtooth fluctuation pattern in the tensile curve. Compared to strip geogrid material, hexagonal-type gabion mesh effectively increase the tensile strain withstood by the soil and increase the tensile load of the soil.

Failure is observed at the nodes of the plastic geogrid mesh during the tensile process, which indicates stress concentration in the node sections. The fiberglass geogrid rib fractures in the middle section when subjected to tensile loading. The edge wires of the gabion mesh are compressed toward the center of gabion mesh during the tensile process. There is no stress concentration due to the elastic–plastic characteristic of gabion steel wire.

The standard linear three-element model, the nonlinear three-element model, and improved Kawabata model can all well reflect the tensile behavior of six different types of geotechnical reinforcement materials. The simulation effect of the standard linear three-element model and the nonlinear three-element model is better than that of the improved Kawabata model for plastic geogrid mesh and fiberglass geogrid mesh. However, the improved Kawabata model can better capture the tensile behavior of gabion mesh.

## Figures and Tables

**Table 1:** Main mechanical parameters of reinforcements based on results of tensile tests.

Type of Reinforcement Material	Length/mm	Width/mm	Maximum Tensile Load/kN	Tensile Force at 2% Tensile Strain/kN/m	Tensile Force at 5% Tensile Strain/kN/m)	Maximum Tensile Strength/kN/m	Maximum Tensile Strain/%
Plastic geogrid mesh	530	80	3.19	25.7	33.4	39.8	12.70
Fiberglass geogrid mesh	350	70	6.90	48.5	82.1	98.9	7.23
Gabion mesh	280	450	19.40	5.7	7.7	43.5	26.58
Plastic geogrid rib	340	/	1.24	/	/	310 **	16.63
Fiberglass geogrid rib	470	/	3.14	/	/	314 **	5.30
Gabion steel wire	360	/	2.72	/	/	475 **	11.52

Note: ** represents MPa.

**Table 2:** Main simulation parameters of different reinforcement materials.

Type of Reinforcement Material	Standard Linear Model				Nonlinear Model			Improved Kawabata Model		
	*k*/ mm·s^−1^	*η*/ MPa·s·mm^−1^	*E*_2_/ MPa	*E*_1_/ MPa	*η*/ MPa·s·mm^−1^	*E*_0_/ MPa	*b*/ MPa	*f*_max_/ MPa	*L*_t_	*W*_t_/ MPa
Fiberglass geogrid rib	3.22	3.22	−62.02	40.25	32.68	62.95	4.6	9.36	1.0	0.93
Plastic geogrid rib	4.0	4.75	−7.91	3.39	11.09	10.53	0.12	0.53	1.0	0.02
Gabion steel wire	40.31	−9.78	−150.5	−127.5	97.14	6.27	46.63	100.3	0.82	57.32

**Table 3:** Simulation parameters of different reinforcement meshes.

Type of Reinforcement Material	Standard Linear Model				Nonlinear Model			Improved Kawabata Model		
	*k*/ (mm·s^−1^)	*η*/ (kN·m^−1^·s·mm^−1^)	*E*_2_/ kN/m	*E*_1_/ kN/m	*η*/ (kN·m^−1^·s·mm^−1^)	*E*_0_/ kN·m^−1^	*b*/ kN·m^−1^	*f*_max_/ kN·m^−1^	*L*_t_	*W*_t_/ kN·m^−1^
Gabion mesh	−453.9	0.0002	1.19	−0.02	−606.5	0.9	0.029	0.07	0.9	0.003
Plastic geogrid mesh	5.44	5.46	−8.93	0.26	5.79	9.18	0.01	6.38	1.03	2.6
Fiberglass geogrid mesh	3.32	3.32	−21.9	11.68	12.21	27.89	1.17	40.02	1.05	51.13

## Data Availability

The original contributions presented in the study are included in the article, further inquiries can be directed to the corresponding author.
